# The influence of physical literacy of student with different obesity levels on physical fitness: the mediating effect of MVPA

**DOI:** 10.3389/fpubh.2024.1463108

**Published:** 2024-10-04

**Authors:** Wenjing Yan, Mingjian Nie, Ruisi Ma, Qi Guo, Hongjuan Li

**Affiliations:** ^1^School of Physical Education, Shanxi Normal University, Taiyuan, Shanxi, China; ^2^School of Sport Science & Key Laboratory of the Ministry of Education of Exercise and Physical Fitness, Beijing Sport University, Beijing, China; ^3^School of Physical Education, Jinan University, Guangzhou, Guangdong, China; ^4^Department of Prenatal Diagnosis Center, Women and Children’s Hospital of Chongqing Medical University; Department of Prenatal Diagnosis Center, Chongqing Health Center for Women and Children, Chongqing, China

**Keywords:** perceived physical literacy, physical activity, obesity, meditation, college & university students

## Abstract

**Background:**

Perceived physical literacy (PL) is a potential factor for improving health and physical activity, but the relationship between PL and physical fitness (PF) among college students with different obesity levels has not yet been determined.

**Purpose:**

The aim of this study was to explore the correlation between PL, moderate-to-vigorous physical activity (MVPA), and PF among college students with different obesity levels.

**Methods:**

We recruited Chinese university students to complete a questionnaire and conducted a survey using the Perceived Physical Literacy Instrument Scale (PPLI-SC) and the International Physical Activity Questionnaire Short Form (IPAQ-SF). The physical fitness test data were used for data analysis. The direct and indirect impacts were analyzed using Pearson correlation and the SPSS Hayes process macro (Model 4).

**Results:**

The study surveyed 909 boys and 1,668 girls for a total of 2,577 valid questionnaires. Similarly, the MVPA and perceived PL of boys were significantly greater than those of girls (*p* < 0.01), but the opposite was true for the PF score. The correlation analysis showed that MVPA, perceived PL, and PF were significantly correlated (*p* < 0.01). For normal weight student, the results showed that the direct effect of PL on PF was still statistically significant (*β* = 0.076, *p* < 0.01) after adding MVPA; MVPA had a positive effect on PF after controlling for perceived PL (*β* = 0.055, *p* < 0.05). Perceived PL has a positive effect on MVPA (*β* = 0.123, *p* < 0.01). The mediating effect of MVPA was notably significant, with a mediation effect percentage of 7.9%. However, it has been found that among the underweight population, there is an absence of a significant relationship between PL, PF, and MVPA (*p* > 0.05). Contrarily, in the overweight and obese groups, the mediating role of MVPA in the relationship between PL and PF was not statistically supported (−0.002, 0.033).

**Conclusion:**

For normal weight student, perceived PL has a positive impact on PF and can also be promoted by increasing the pathway of MVPA. For both underweight individuals and obese populations, further investigation is needed into methods for promoting PA and improving PF.

## 1 Introduction

Physical activity (PA) is beneficial for the development of physical and mental health (1, 2). Although an increasing number of physical activity strategies have been proposed, the phenomenon of physical activity is globally widespread (3). A sedentary lifestyle has evolved into a public health issue (4). One quarter of adults worldwide are unable to meet the global recommendations on physical activity levels (3). Significant changes have also taken place in college students’ physical activity (5). COVID-19 has led to a significant increase in sedentary behavior and a significant decrease in college students’ physical activity (6, 7). With increasing sedentary behavior, the stress, anxiety, and depression of college students significantly increase (8, 9). To address this challenge, it is necessary to improve the quality and quantity of physical activity participation throughout the entire life cycle to maintain a healthy and active lifestyle.

The importance of physical literacy (PL) has attracted widespread attention from the international community, and many countries have developed intervention models and policies based on PL to improve PA and health (10, 11). The World Health Organization has identified PL as an important component of its action plan to address global public health issues related to physical inactivity (12). PL is a multidisciplinary and comprehensive concept, and the intersection of motor skills, positive emotions, and motivation is the core element needed to ensure positive activity as well as the prerequisite and foundation for individuals to participate in PA throughout their lifetime (13). In recent years, there has been much discussion on the concept of PL, with additional research on the attributes of PL mainly focusing on emotions, physical abilities, cognition, and behavior. At present, the concept proposed by Whitehead has been widely accepted. Whitehead noted that physical fitness refers to the motivation, confidence, physical fitness, knowledge, and understanding to value and take responsibility for lifelong PA (14). Perceived PL plays an important role in developing healthy eating habits among adolescents (15). Individuals with higher levels of perceived PL will have greater confidence and ability to engage in various PAs. A survey targeting Chinese undergraduate students found a significant correlation between perceived PL and PA levels. This study provides a pathway for how enhancing perceived PL can promote PA among Chinese undergraduate students (16). Research has also found a close relationship between university students’ PL and health-related quality of life, with physical activity and subjective well-being playing mediating roles (17). At present, there are few tools available for evaluating the PL of college students (18, 19), and further research is needed to evaluate college students’ PL.

The physical fitness (PF) of college students has always been an important concern. The physical fitness testing for Chinese university students is guided by the “National Standards for Students’ Physical Fitness and Health” established by the Ministry of Education of China. The test items for university students include a 50-meter sprint, sit-and-reach flexibility test, standing long jump, pull-ups (for males), sit-ups (for females), a 1,000-meter run (for males), and an 800-meter run (for females) (20). However, the current situation of PF among college students is not optimistic. Research has shown a negative correlation between students’ physical fitness and anxiety symptoms (21). The home isolation environment during COVID-19 significantly reduced the physical fitness of college students, especially in terms of endurance and flexibility (22). A study revealed that a decrease in PF was observed between 1985 and 2014. With the advancement of urbanization, the physical fitness of both rural and urban students in these areas is decreasing (23). This finding confirms the decrease in PF among college students.

There is clear evidence that Perceived PL can have a positive impact on Moderate-to-Vigorous Physical Activity (MVPA) and health (24). There are positive correlations between PA and cardiovascular health and physical health, and both PA and prolonged sitting can affect PF (25). However, there is limited evidence for the role of physical activity in PL and PF, and it remains to be determined whether physical activity is involved in these pathways. At present, there are many studies on the relationship between the PL and PA, but there is a lack of research on the relationship between the PF and PA (26). There is limited evidence regarding the direct association between the PL and PF among college students with different obesity levels. Therefore, the purpose of this study was to explore the direct correlation between the PL and PF and to verify the role of MVPA among college students with different obesity levels. This study proposes a hypothesis path based on relevant research: Perceived PL positively affects PF, which can also be improved by increasing MVPA.

## 2 Methods

### 2.1 Study design and participants

This study adopted a cross-sectional design where participants were randomly solicited to partake in questionnaire completion. The Perceived Physical Literacy Index for Students (PPLI-SC) (18) was employed to assess the subjective levels of Physical Literacy (PL) among the study cohort, while the International Physical Activity Questionnaire Short Form (IPAQ-SF) (27) was utilized to gage their Physical Activity (PA).Physical health status evaluations were fundamentally rooted in the outcomes derived from a standardized physical fitness assessment tailored for collegiate populations. Additionally, comprehensive demographic data, encompassing gender, age, stature, and body mass, were systematically collected. Prior to engaging with the questionnaire, participants had furnished their informed consent, a process which involved meticulously reviewing an online consent form. Following their explicit agreement with its contents, they proceeded to populate the questionnaire. In contrast, those who withheld consent were automatically redirected away from the study. Participants were recruited randomly through physical education courses at universities to complete the questionnaire. The on-site guidance for filling out the questionnaire was provided jointly by professionals and physical education teachers. The testing period was from June 2023 to June 2024. To ensure rigorous ethical standards, it is emphasized that this research was subjected to and received ethical approval from the Scientific Experiment Ethics Committee of Shanxi Normal University (2024–0501).

### 2.2 Perceived physical literacy of college students

Ma adapted the initial set of 18 questions in the PPLI-SC, refining them into an eight-item instrument specifically calibrated for Chinese college students (18). This compacted version serves as a measure of the perceived PL among Chinese undergraduate students, focusing on four core dimensions: motivation, self-confidence, engagement in physical competitions, and environmental interactions. The instrument utilizes a validated five-point Likert scale, ranging from ‘strongly disagree’ to ‘strongly agree’, allowing for nuanced gradations in responses. The total score is calculated by aggregating the scores across all eight items, thus falling within a range between 8 and 40. A higher cumulative score signifies a more favorable perception of PL, thereby offering a more refined and contextually appropriate metric for assessing PL among this specific population subset.

### 2.3 Physical activity

PA was investigated with the IPAQ-SF. This scale is used to evaluate the frequency and duration of light, moderate and vigorous PA as well as meditation in the past week via seven questions. Moderate-to-vigorous physical activity (MVPA) was performed when 3.0 or more Metabolic Equivalents (METS) were performed. The duration of the questionnaire was measured in hours and minutes, and the activity frequency was recorded in days. The responses are subsequently translated into metabolic equivalents (MET minutes). The MVPA score refers to the sum of moderate and vigorous PA scores. The reliability test of the IPAQ-SF is 0.80 (27).

### 2.4 Physical fitness

Physical Fitness (PF) metrics were sourced from the annual student physical fitness assessments. The comprehensive physical fitness assessment suite consists of eight specialized tests designed to measure various aspects of fitness. Included in this battery are the 50-meter sprint, timed in seconds to gage speed; the sit-and-reach test, measuring flexibility in centimeters; and the standing long jump, assessing explosive power and lower body strength, also quantified in centimeters. Vital capacity, a critical indicator of respiratory health, is calculated as milliliters per kilogram of body weight for all participants regardless of gender. For male students, the regimen includes pull-ups, with the number of repetitions indicating upper body strength; and a 1,000-meter run, timed in seconds to evaluate cardiovascular endurance. Female participants undergo an 800-meter run, similarly timed in seconds to assess aerobic capacity; and a sit-up test, where the quantity of repetitions performed is counted to measure core strength. All physical fitness evaluations are conducted in strict adherence to the performance benchmarks set for college students, with the aggregated results contributing to a holistic fitness score. Students’ physical fitness scores are rated as follows: 90.0 and above are considered excellent, 80.0 to 89.9 are good, 60.0 to 79.9 are passing, and below 59.9 are failing.

### 2.5 Data analysis

SPSS 22.0 software was used to establish a database for analysis of the results. Count data are represented by “rate” (%), and measurement data are represented by “mean ± standard deviation” (𝑥 ®± s). According to the BMI classification in China, underweight is defined as BMI < 18.5, normal weight as BMI = 18.5–24, overweight as BMI = 24–28, and obesity as BMI ≥ 28. The SPSS Hayes process macro (Model 4) was used to analyze direct and indirect effects. We used Pearson correlation and linear regression analyses to evaluate the relationships between indicators. We used an independent sample t test and Chi-square test for difference analysis. The significance level was defined as *p* < 0.05, and the significance level was defined as *p* < 0.01.

## 3 Results

### 3.1 Student characteristics

There are 2,577 effective persons in this survey. The number of boys participating in the study is 909, which constitutes 35.36% of the total sample size. Concurrently, the number of girls involved in the research is 1,668, and they also make up 35.3% of the entire population under consideration (Table 1).

**Table 1:** Statistics of basic student information.

	Boy *N* = 909 (35.3.6%)	Girl *N* = 1,668 (35.3%)
Age (years)	21.15 ± 1.35	20.96 ± 1.23
High (cm)	176.65 ± 6.27	163.19 ± 5.63
Weigh (kg)	72.58 ± 13.37	56.59 ± 8.44
Bmi (kg/m2)	23.21 ± 3.82	21.23 ± 2.83
Underweight	135	5.24%
Normal	1914	74.27%
Overweight and obese	528	20.49%
Grade		
1	854	33.14%
2	618	23.98%
3	656	25.46%
4	449	17.42%

### 3.2 Difference analysis

The PL and MVPA of boys were significantly greater than those of girls, while the PF of girls was greater than that of boys. The proportion of students excelling in PF is marginal at merely 0.31%, whereas the failure rate surges to 22.62%. Notably, female students exhibit a significantly lower failure rate of 16.13%, markedly contrasting with their male counterparts’ rate of 34.54%. These disparities underscore the gender divide in physical fitness performance among university students (Table 2).

**Table 2:** Analysis of gender differences.

	Boy *N* = 909 (35.3.6%)	Girl *N* = 1,668 (35.3%)	
MVPA	2708.81 ± 2519.26	1964.85 ± 2019.93^**^	
Confidence and physical competitions	11.47 ± 3.18	10.51 ± 2.92^**^	
Environmental interactions	11.68 ± 3.00	11.23 ± 2.68^**^	
Motivation	7.98 ± 2.01	7.65 ± 1.79^**^	
PL	31.13 ± 7.75	29.40 ± 6.75^**^	
50-meter sprint (measured in seconds)	7.94 ± 0.85	9.82 ± 0.81^**^	
Sit-and-reach flexibility test (documented in centimeters)	12.22 ± 8.60	17.19 ± 6.87^**^	
Standing long jump (also measured in centimeters)	215.02 ± 24.55	161.88 ± 18.01^**^	
Vital capacity	4161.35 ± 802.46	2761.89 ± 542.50^**^	
Pull-up (recorded as the number of repetitions performed)	5.36 ± 5.51		
1,000-meter run (recorded in seconds)	278.76 ± 52.88		
800-meter run (timed in seconds)		259.63 ± 37.74	
Sit-up test (counted in the number of repetitions completed)		33.67 ± 7.20	
PF	63.62 ± 12.10	69.26 ± 10.03^**^	
PF Classification			
Excellent	8	0.31%	
Good	193	7.49%	
Passing	1793	69.58%	
Failing	583	22.62%	
			All
Passing	595	1,399	1994
Failing	314	269	583
All	909	1,668	2,577
Ratio	34.54%	16.13%^**^	22.62%
Pearson Chi-Square	113.996		

**There is a statistically significant difference between males and females.

There was no significant difference in the PL and MVPA among individuals with different body shapes, but the PF of students with normal weight was significantly higher than that of underweight, overweight, and obese students (Table 3).

**Table 3:** Analysis of body shapes differences.

Category	Underweight (135)	Normal (1914)	Overweight and obese (528)	F	*p*
PL	30.39 ± 7.01	30.04 ± 7.12	29.80 ± 7.36	0.426	0.653
MVPA	2490.70 ± 2387.07	2212.40 ± 2250.38	2213.83 ± 2146.74	0.988	0.372
PF	65.20 ± 10.10^b^	69.56 ± 9.77^a^	59.47 ± 12.35	198.996	0.000

a Normal weight are significantly higher than those who are obese and underweight.

b Underweight students are significantly higher than obese students.

### 3.3 Relationships between variables

The direct effect refers to the immediate influence of one variable (the independent variable) on another variable (the dependent variable). The indirect effect occurs when the independent variable influences the dependent variable through one or more mediating variables. A total effect is the sum of direct and indirect effects. Full mediation refers to the situation where the effect of the independent variable (IV) on the dependent variable (DV) is entirely transmitted through one or more mediating variables. In other words, the IV has no significant direct effect on the DV, and its influence is entirely realized through the mediating variables. Partial mediation occurs when the effect of the IV on the DV is only partially transmitted through one or more mediating variables, while the IV still has a significant direct effect on the DV. In this case, the mediating variables explain only part of the effect of the IV on the DV, rather than the entire effect.

The findings revealed significant associations between Perceived PL and PF (*r* = 0.093, *p* < 0.01), PL and MVPA (*r* = 0.164, *p* < 0.01), as well as between MVPA and PF (*r* = 0.066, *p* < 0.01). These observed correlations facilitated the construction of a mediation model to probe the underlying mechanisms linking PL and PF. To substantiate this mediation, a mediation effect analysis was conducted, involving the formulation of three regression equations, as detailed in Table 4.

**Table 4:** Variable regression analysis and mediating effect test.

	Equation	Effect	F	P	*β*	*t*	*p*		
Underweight	PF = cPL + e_1_	c	5.117	0.001	0.125	1.054	0.294		
	MVPA = aPL + e_2_	a	5.141	0.001	0.115	1.392	0.166		
	PF = c’PL + bMVPA+e_3_	c’	4.374	0.001	0.075	0.120	0.907		
		b			0.102	1.161	0.248		
Normal	PF = cPL + e_1_	c	57.073	0.000	0.083	3.820	0.000		
	MVPA = aPL + e_2_	a	29.631	0.000	0.123	5.514	0.000		
	PF = c’PL + bMVPA+e_3_	c’	47.013	0.000	0.076	3.484	0.001		
		b			0.055	2.481	0.013		
		Effect	se	*t*	*p*	LLCI	ULCI	c’_cs	Effect
	Total effect of X on Y	0.114	0.030	3.820	0.000	0.056	0.173	0.083	
	Direct effect of X on Y	0.105	0.030	3.484	0.001	0.046	0.164	0.076	92.1%
	Indirect effect(s) of X on Y	Effect	se	LLCI	ULCI				
	MVPA	0.009	0.005	0.001	0.020				7.9%
Overweight and obese	PF = cPL + e_1_	c	26.300	0.000	0.209	5.167	0.000		
	MVPA = aPL + e_2_	a	11.606	0.000	0.223	5.242	0.000		
	PF = c’PL + bMVPA+e_3_	c’	21.573	0.000	0.195	4.698	0.000		
		b			0.064	1.545	0.123		
		Effect	se	*t*	*p*	LLCI	ULCI	c’_cs	Effect
	Total effect of X on Y	0.352	0.068	5.167	0.000	0.218	0.485	0.209	
	Direct effect of X on Y	0.328	0.070	4.698	0.000	0.191	0.465	0.195	
	Indirect effect(s) of X on Y	Effect	se	LLCI	ULCI				
	MVPA	0.014	0.009	−0.002	0.033				

*Control for gender, age and grade. The independent variable X had an impact on the dependent variable Y. If X affected Y by influencing variable M in addition to directly influencing Y, then M was called the intermediate variable. This was called the mediation effect. Term c represents the total effect of X on Y. Term a represents the effect of X on M, c’ represents the effect of X on Y after controlling M, and b represents the effect of M on Y after controlling X.

**Figure 1:**
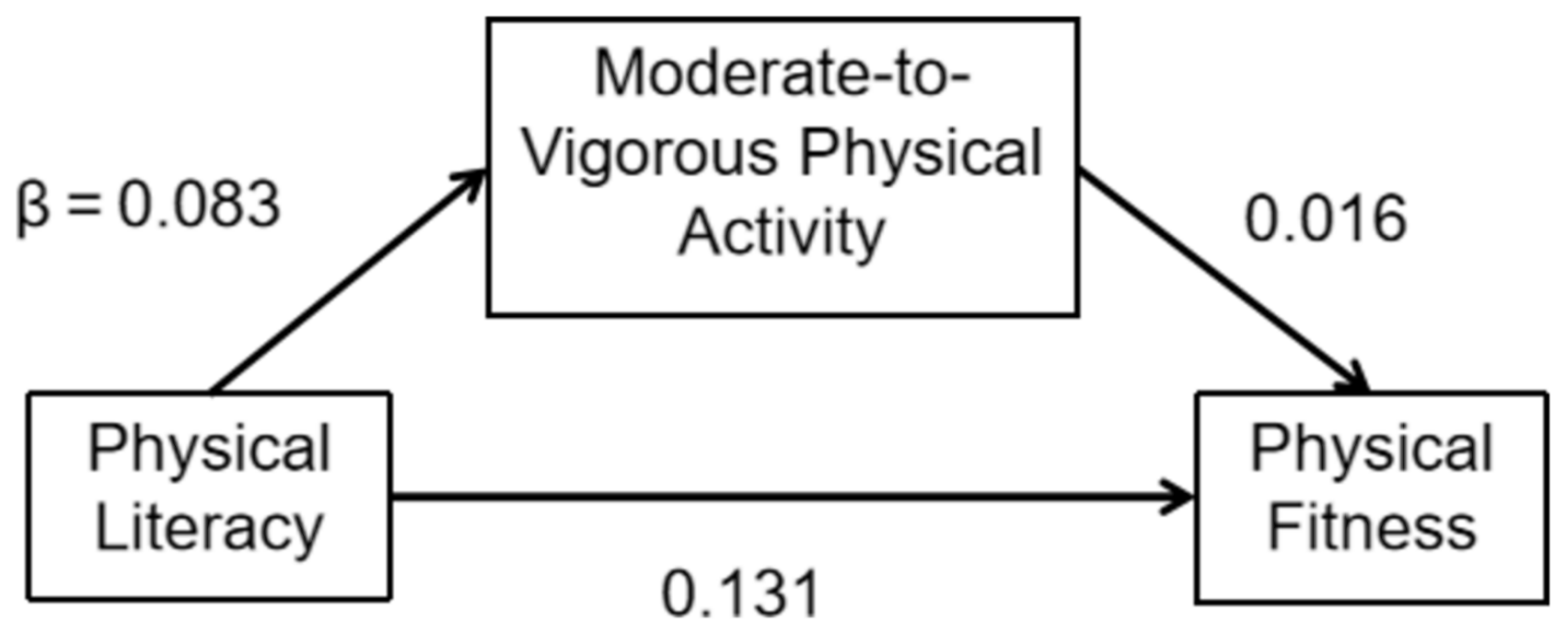
From effects of PL on PF mediating effects of MVPA(normal-weight).

For normal-weight individuals, however, PL demonstrated a statistically significant positive influence on MVPA (*β* = 0.115, *p* < 0.01), as well as on physical fitness (*β* = 0.131, *p* < 0.01; Figure 1). This indicates that there exists a certain degree of direct association between students’ PL engagement and their physical fitness, suggesting that students participating in PL tend to have better fitness levels. The mediating effect of MVPA was notably significant, with a mediation effect percentage of 7.9%. This implies that PL participation among normal-weight students can indirectly enhance their physical fitness by boosting their MVPA levels. In summary, the mediation analysis indicated that MVPA partially mediates the relationship between PL and PF, providing empirical evidence for the intricate interplay among these constructs, which is essential for understanding and potentially improving the physical literacy and fitness of the studied population.

Among underweight individuals, the impact of PL on MVPA was not statistically significant (*β* = 0.155, *p* = 0.166), and similarly, the direct effect of PL on physical fitness was also not significant (*β* = 0.075, *p* = 0.907). Based on these findings, there is no significant direct relationship between PL, MVPA, and physical fitness for underweight individuals.

Regarding overweight and obese individuals, a statistically significant positive correlation was found between PL and MVPA (*β* = 0.223, *p* < 0.01), and PL exerted a significant direct positive effect on PF (*β* = 0.209, *p* < 0.01). Nonetheless, the mediating effect of MVPA on the relationship between PL and physical fitness for this group was not statistically significant (−0.002, 0.033).

## 4 Discussion

This study explored the relationships between PL and MVPA and between PL and PF, providing effective evidence that perceived PL promotes physical health. The results indicate that normal-weight college students’ perceived PL and MVPA can positively affect their PT, and PL can also improve their PF by increasing the impact of MVPA. However, it has been uncovered that within the underweight population, no substantial correlation emerges between PL, PF, and MVPA. Among the overweight and obese cohorts, the hypothesized mediating effect of MVPA on the linkage between PL and PF fails to attain statistical significance. Not only should we pay attention to the physical fitness of overweight and obese individuals but also to the proportion of students who are underweight.

After students reach the higher education stage, their PAs undergo significant changes (5). The higher education stage is an important period during which students receive PL interventions and actively participate in physical activities. It is also important for students to passively accept basic knowledge and maintain a positive attitude toward physical activities in an organized manner (28). PL has attracted increasing research attention in the promotion of physical education and PA participation (29). Kwan et al. (28) found that interventions based on PL can effectively reduce the decrease in PA observed by college students in the first year of school while also helping to maintain their physical health. Therefore, universities remain a potential environment for supporting the development of PLs and establishing positive PA patterns. For college students, promoting PL should be identified as the primary goal of physical education in universities, and adopting health interventions guided by the concept of PL is crucial for improving students’ health.

The multifaceted determinants of college students’ physical fitness encompass a broad spectrum of factors. Externally, elements such as teaching content, organizational structures, instructor competence, assessment methodologies, infrastructure provisions, and other pragmatic considerations play pivotal roles in shaping students’ overall physical fitness levels (30). The environmental context, embodying both the physical layout and cultural ambiance of educational institutions, is paramount. Specifically, universities offer a tailored physical setting conducive to fostering positive physical activity (PA) behaviors among students, facilitated by campus facilities, residential arrangements, class structures, and extracurricular clubs that foster interpersonal dynamics (28). Complementing the physical milieu, educational institutions also establish a pedagogical ecosystem for physical education, aimed at skill instruction, knowledge dissemination, and nurturing a lifelong commitment to PA engagement (31).Empirical investigations abound regarding the correlations between PF and PA, as well as the intricate linkages between PL and PA participation. Whitehead emphasize that the promotion of PL is instrumental in sustaining PA across the lifespan (32). Davids et al. further assert that PL enhancements lead to a higher caliber and frequency of lifelong PA engagement (33). Yan et al. (24) extend this understanding by demonstrating PL’s predictive capacity for both PA and sedentary behavior. This accumulating body of research, complemented by findings from the present study illustrating a robust connection between college students’ perceived PL and PF, underscores PL as a pivotal determinant influencing the healthy evolution of PF. Moreover, it reinforces the notion that exercise behavior operates as a vital mediator in the intricate interplay between PL and PF, thereby validating the centrality of PL-driven interventions in fostering healthier lifestyles among college students.

Research addressing the physical activity patterns among underweight individuals remains limited in comparison to the extensive literature on obesity. Our study reveals that underweight individuals report higher levels of MVPA and PL relative to those with normal weight and obesity, albeit these differences lack statistical significance. Of note, PF was significantly higher in students with normal weight when contrasted against both underweight and obese populations. These observations concerning MVPA diverge somewhat from previous research asserting that adolescents of normal weight demonstrate significantly greater physical activity compared to their underweight or obese peers (34). We speculate that the smaller sample of underweight participants in our study might have impacted these results. Notably, separate studies have emphasized how underweight students often practice healthier eating behaviors and spend less time in sedentary activities, whereas those with high body fat percentages are associated with less healthy diets and increased sedentarism (35). Moreover, our research uncovers no statistically significant links between PL, MVPA, and PF among underweight individuals, suggesting a complex relationship dynamic within this group. Intriguingly, within the obese cohort, we did not observe a mediating effect of MVPA on PL. These insights emphasize the necessity for developing targeted intervention strategies that cater specifically to the needs and contexts of underweight populations to augment physical activity engagement and overall physical health. Conversely, for obese individuals, interventions focused on enhancing physical literacy still hold potential for stimulating increases in physical activity and, by extension, physical fitness. Consequently, there is a pressing need for further exploration into efficacious intervention methodologies, especially tailored to underweight populations, to comprehensively tackle the diverse range of body mass indices and their implications for population health.

There are currently many methods and strategies for promoting PA and health among college students. There is evidence to suggest that school-based physical education is a good opportunity to promote and develop PL (36). Short-term health interventions can have a positive impact on the health behavior of college students, which may lead to positive health performance during the participation period and facilitate the transition from high school to university (37). In traditional strategies for promoting sports health, emphasis is placed on intervention in knowledge and physical abilities, but emotional dimensions are actually very important factors (26). Cowley et al. (38) found that the movement disorders of students after the age of 16 come from traditional physical education courses that prioritize exercise ability. Therefore, in the new era, college students should pay attention to the concept of PL. PL provides a new concept for college students’ PA and health promotion interventions, and we can promote lifelong habits related to physical activity by providing them with more opportunities for various activities (39). By promoting PL and PA measures, students can master knowledge and skills related to PL, cultivate their awareness of lifelong exercise, and enhance their motivation to participate in physical activities.

## 5 Limitations and future directions

This study’s reliance on self-reported PA & PL data invites recalled bias and potential overestimation. Gender imbalance, favoring females, limits generalizability due to random sampling. Moreover, it solely examines the PL-PF and PA-PF relationships, omitting other influential variables, potentially simplifying the actual complexity. Future research should adopt objective measurement tools to alleviate reporting biases and strive for gender parity in sampling. Future research could also place more emphasis on the differences between athletes and non-athletes, as well as the distinctions between healthy students and those with disabilities. It should expand to investigate a broader spectrum of factors affecting PF, acknowledging the intricate web of influences beyond PL and PA alone. This nuanced exploration will facilitate the development of advanced models informing interventions to promote holistic physical health.

## 6 Conclusion

This study confirmed that normal-weight college students perceive that PL can enhance PF, and MVPA is an important mediating factor in this relationship. Moreover, physical health can be promoted by enhancing the MVPA. However, it has been found that among the underweight population, there is an absence of a significant relationship between PL, PF, and MVPA. In the overweight and obese groups, the mediating role of MVPA in the relationship between PL and PF was not statistically supported. Therefore, universities should develop the concept of developing PL when researching strategies to promote the physical health of normal-weight college students. For underweight and overweight/obese individuals, there is a clear necessity to delve into distinct strategies for physical activity interventions, a pursuit that may contribute significantly to improved health management and understanding within these populations. Our research findings provide a theoretical basis for developing strategies to improve the health of college students by promoting positive exercise behavior and PL.

## Data Availability

The original contributions presented in the study are included in the article/supplementary material, further inquiries can be directed to the corresponding authors.
